# 

**Published:** 1894-01-13

**Authors:** 


					fBE HOSPITAL. c 7   ^
X A \rrf a -t?t-r 1 Omrf "I*
PRICE TWOPENCE,. ?ss^ WIS f ^5^ WITH EXTRA NURSING SUPPLEMENT
Per Annum, 8s. 6d., post free.
January 13th, i
? PRICE TWOPENCE. yA
No. 381, Vol. XV.?Jan. 13th, 1894. ^|T
Registered at the General Post Office as a Newspaper for
transmission in the United Kingdom and Ahmad 1 c??
/ f^c?i8t0ped at the General Post Office as a Newspaper for |L JTA jm Monthly Farts, 6d.; post free, 8d.
tranamission in the United Kingdom and Abroad.] Ditto per Annum, 8s? post #eeTV
W &k
ME
RW1CH HOSPl.TAX
No. 381. Vol. XV. WITH EXTRA NURSING SUPPLEMENT. Saturday,
Jawuary 13th, 1894.

				

